# Supplementary material for: Clade-specific elemental signatures across an Early Triassic marine fauna pave the way for deciphering the affinities of unidentifiable fossils
Source: PLoS One. 2025 Aug 13;20(8):e0329498. doi: 10.1371/journal.pone.0329498 (PMC12349085; doi:10.1371/journal.pone.0329498)
Supplement: S1 Data — S1 File. This file contains: Data S1 - Pictures of the analysed samples exported from PyMCA with the studied zone highlighted by the shaded area. Data S2 - Mean µ-XRF spectrum of each studied zone. The data presents the mean value of photons measured per pixel (“counts” column) per energy level (“energy” column) over the sampled area. Data S3 - Mean µ-XRF spectrum of each studied zone regrouped per- specimens such as to be statistically analysed using R software S2 File. This document contains Figs S1, S2, Table S1, and supplementary text regarding the µXRF spectra morphological descriptors approach and the data acquisition setups. S3 File. This document is the R data analysis script. (DOCX) [file pone.0329498.s001.docx]

**Supporting information**

**All supporting information for this study is available in the Dryad Digital Repository:** https://doi.org/10.5061/dryad.2rbnzs7v7**.**

**-S1 File. “S1 File_raw data_Smith et al”.** This file contains: Data S1 - Pictures of the analysed samples exported from PyMCA with the studied zone highlighted by the shaded area. Data S2 - Mean µ-XRF spectrum of each studied zone. The data presents the mean value of photons measured per pixel ("counts" column) per energy level ("energy" column) over the sampled area. Data S3 - Mean µ-XRF spectrum of each studied zone regrouped per- specimens such as to be statistically analysed using R software

**-S2 File.** **“S2_File_Supplementary Materials_Smith et al”.** This document contains figures S1, S2, Table S1, and supplementary text regarding the µXRF spectra morphological descriptors approach and the data acquisition setups.

**-S3 File. “S3_File_R script_Smith et al”.** This document is the R data analysis script.
