# Supplementary material for: Clade-specific elemental signatures across an Early Triassic marine fauna pave the way for deciphering the affinities of unidentifiable fossils
Source: PLoS One. 2025 Aug 13;20(8):e0329498. doi: 10.1371/journal.pone.0329498 (PMC12349085; doi:10.1371/journal.pone.0329498)
Supplement: Attachment — Submitted filename: Response to Reviewers.docx [file pone.0329498.s003.docx]

***Dr. Christopher P. A. Smith***

CR2P, UMR 7207, CNRS,

Muséum national d’Histoire Naturelle

8 rue Buffon, 75005 Paris, France

Email: [christopher.smith@mnhn.fr](mailto:christopher.smith@mnhn.fr)

To: Editorial office of

**PLOS ONE**

June 24, 2025

Dear Editor,

Please find enclosed the revised version of our manuscript ***Clade-specific elemental signatures across an Early Triassic marine fauna pave the way for deciphering the affinities of unidentifiable fossils*** by Christopher. P. A. Smith, Pierre Gueriau, Mathieu Thoury, Sebastian Schöder, Emmanuel Fara, Arnaud Brayard (Manuscript number PONE-D-25-05930)

As requested, you will find two versions of the main text of the manuscript:

- One in which the corrections are incorporated, entitled:

“Smith et al _revised_MS_PONE_D_25_05930.docx”.

- One with the follow-up of the corrections, entitled:

“Smith et al _revised_MS_PONE_D_25_05930_track_changes.docx”.

*•This manuscript has been revised after careful consideration of all the comments and suggestions made by the two reviewers.*

*• This revised manuscript comprises 8370 words in total (not including the 95 references), and 9 figures (provided separately), and 1 table (included in the main text). The Supplementary Material comprises 1 text document (with three figures, one table, and five additional references), three data files, and one R software script.*

*• Supplementary Material will be made available through Dryad repository on acceptance.*

*• Regarding Figure 1, it is modified from Brayard et al. (2017) and Smith et al. (2021), both of which are published under a CC BY 4.0 license. Therefore, there is no copyright issue.*

*• This manuscript is original and is not under consideration elsewhere, including the internet.*

*• All the studied material is hosted in a public institution (Université Bourgogne Europe, Dijon, France).*

We sincerely thank the editor for his editorial work, and both reviewers for their thorough, in-depth, and particularly constructive reviews. We are especially grateful for the high quality of the feedback, as we understand that this manuscript spans several fields (statistics, geochemistry, palaeontology), which may not be everyone's area of expertise.

We have carefully considered all the comments and suggestions made by the two reviewers. In addition, we made some minor modifications to the statistical analyses to enhance the rigor of our approach. Importantly, these changes did **not** alter the overall results.

Among the adjustments:

- Homologous data (i.e., data from the same studied zone) acquired using different setups were merged into single data points to avoid over-representation of these zones.
- The LDA models were recalibrated using 98% of the PCA variance (instead of 95%), which strengthened the LDA models and improved their leave-one-out test accuracy by approximately 5%.

As a result, the best-performing LDA model now achieves around 90% correct classification.

Please find below our responses to each comment, highlighted in blue (see also tracked changes in the revised manuscript).

**Answers to reviewer 1 details:**

Smith and co-authors present an innovative exploratory investigation of paleontological applications of synchrotron micro-X-ray fluorescence utilizing a diverse set of metazoan body fossils and associated sediments.

Notably, the authors found that across three rounds of data collection, instrumental parameters exerted a strong influence on spectral results, complicating comparison of datasets across instrumental setups. To address this the authors employ a correction on their multivariate data using samples scanned across multiple setups to serve as calibration.

Following this correction procedure the authors show that the multivariate and cluster analysis broadly differentiate fossil spectra from sediment spectra although certain fossil samples show “sediment like” values or trends possibly due to their mineralogy and or preservation state. Conversely, the authors dismiss site-specific variation as a major influence on their multivariate analyses. Linear discriminant analysis shows success in distinguishing large-scale taxonomic groups within their dataset, indicating potential for taxonomic classification using this technique although this is not directly tested or demonstrated in this investigation.

Finally the authors investigate the spectral regions (corresponding to specific elements) which contribute to observed variation within sediment samples and which discriminate among taxonomic groups, finding Ca, Sr, Fe and some other elements as particularly important. They speculate about factors that might account for taxonomic differences in spectral measurements and elemental composition.

Overall these are interesting and apparently novel results presented in a generally well-written paper. We find that some results are slightly overstated or difficult to interpret, while other potentially interesting results are prematurely dismissed or obscured by the study design. We also wonder how the “pseudo-replication” of collecting multiple spectra from each specimen might skew the results or amplify the LDA classification rate. We suggest some possible remedies to address these concerns or additions/clarifications that would be helpful:

**Dismissal of influence of site.** Based on the results shown in 4A, the authors dismiss site-to-site differences as a significant factor in their dataset. The authors state this relatively clearly in their discussion (Lines 513-517) but we think they could use more nuance when initially presenting the results starting at Line 277. We agree that site does not have an overwhelming effect, the best sampled site (PC) effectively spans the entire PC1 v PC2 plot and overlaps with all other sites. However the sites with fewer samples tend to cluster together or align, although this effect may be exaggerated by the smaller sample sizes, variable taxonomic sampling and pseudoreplication of the data.

→ Thanks for this comment. Indeed, the original manuscript presented the interpretation of site influence too assertively. Based solely on visual inspection of the PCAs, we agree that it is not possible to definitively rule out a site effect. The absence of a clear site- clustering (no matter the number of clusters sought and the method used) does suggest that such effect is limited. We nevertheless acknowledge this is not equivalent to demonstrate no influence at all.

Following this comment, we have revised the entire manuscript to present a more nuanced interpretation of potential site effects.

It is however worth clarifying that the LDA model remains site-independent. By design, LDA seeks to maximize discrimination according to the target grouping (in our case, taxonomy), and thus downplays variation unrelated to that axis. Site could only significantly influence the LDA if clade and site were confounded—for example, if each clade were predominantly sampled from a single site. This is not the case in our dataset. The same rationale applies to potential confounding with fossil mineralogical nature or preservation type.

**Influence of preservation mode & primary mineralogy.** The authors include specimens showing a variety of preservation modes: calcitic body fossils, poorly preserved molds with remnant shell material, carbonaceous films (possibly associated with biomineralized remains of differing mineralogy) and phosphatized remains (presumably including a mix of mineralized and non-mineralized tissues). The authors draw attention to the fact that spectra from calcitic echinoderms and poorly preserved ammonoids resemble in some respects the trends observed in the calcareous sediments on the PCA plot and perform a second PCA analysis to explore this tendency.

Otherwise, the authors seem to diminish the roles of primary mineralogy or preservational mode on the spectra shown in their PCA plots. This may be the case but it is a bit difficult to discern because the points on the PCA plots are not distinguished by preservation mode or biomineral geochemistry.

→ This is partly right. We favoured presenting preservation mode and fossil mineralogy in the main text and Supplementary Table S1 rather than in the figure itself, as the PCA plots already convey a considerable amount of information. Our reasoning is that fossils from the same clade can exhibit different preservation modes and mineralogies, and conversely, fossils from different clades can share similar preservation characteristics. This decoupling suggests that clade and preservation mode/fossil mineralogy are somehow independent variables (with the exception of echinoderms, which are typically calcitic). Similarly to site-specific influences, we do not observe a clear clustering based on preservation- or mineralogy-types in the PCA plots.

While we did conduct an additional PCA_1_ where point shapes represent preservation mode/fossil mineralogy (see below), we ultimately chose not to include it in the main text as this figure can complexify the message to the reader, especially since the limited influences of preservation mode/fossil mineralogy are already supported by the intra- and inter-group preservation mode/fossil mineralogy. We think this maintains clarity without compromising scientific transparency.

As noted below, the inclusion of sediment spectra may obscure more subtle distinctions within the fossil data and we suggest exploring PCA, clustering and LD analysis on fossil spectra only to better understand how primary biomineralization, preservation mode, and taxonomy influence the grouping of fossil spectra.

→ Yes, this was indeed explored. We performed both PCA and LDA analyses on fossil spectra alone, excluding sediment data (see figure below). The results were broadly similar to those obtained when sediments were included. For example, the leave-one-out cross-validation score for the LDA performed on fossil spectra alone at the high-level clade grouping (with 98% of the PCA variance) was approximately 91%, compared to 89% for LDA_3_ that is presented in the manuscript. While LDA is effective at distinguishing predefined groups, it is less suited for interpreting the influence of underlying variables, particularly when applied to PCA-transformed data rather than raw spectra, and when dealing with a large number of groups. As such, performing LDA on fossil data alone does not yield substantially new insights.

Finally, from a conceptual standpoint, sediment spectra remain a relevant group to discriminate. Excluding them from the analysis would overlook part of the investigation and reduce the completeness of the comparative framework. For these reasons, we opted to retain the sediment data in the analyses presented.

In their discussion (lines 592-609) the authors frame some interesting hypotheses about these questions, but they could perform some initial testing of some of these ideas using the data they have, but this opportunity goes unrealized here.

→ We agree that further investigations to test these hypotheses would indeed be valuable. However, this is beyond the scope of the present study, which is intended primarily as a proof of concept to demonstrate that clade-specific geochemical signals can be used to discriminate between fossil groups. In this work, our main objective is to establish the feasibility of this approach rather than to exhaustively test all potential underlying mechanisms.

At any rate, we think it would be helpful for the reader to include a table in the beginning of the paper which outlines the key features of their sample dataset. This could compare sample size across taxonomic group (fish, shrimp, lobster, etc) vs. location, original mineralogy, preservation type or quality.

→ As mentioned in the main text, a table is available in supplementary material, as the manuscript is already dense. This is ultimately an editorial decision and we can integrate this table in the main text if reviewers and/or editors truly deem it necessary.

**Combining sediment and fossil spectra**. Throughout their investigation the authors include spectra from a diverse range of fossils as well as closely associated sediment samples (Fig 2A). This allows them to make some interesting comparisons across sediment and fossil data, noting that the sediment spectra tend to follow a continuous narrow distribution across their PCA plot, whereas fossil data follow different trends and are more patchily distributed across PCA space. Clustering analysis generally shows some ability to separate fossils and sediment spectra, although with some exceptions and caveats and with some clustering techniques working better than others. This is all interesting and could have interesting applications e.g., for identifying putatively biotic/abiotic structures, pseudofossils etc.

Shared PCA tendencies or clustering between fossil specimens and their associated sediment samples could speak to the influence of lithology, site etc. on the overall pattern. It appears that values from specific sites tend to show similar values or trends across one or both PC axes on Fig 2A (also noted above). But it is not possible to see which sediment values correspond with which fossil specimens, a version of the PCA plot that shows sediment/fossil spectra linked or grouped could be interesting, although it could also be visually difficult to disentangle. Perhaps it is worth including something like this in the supplement.

→ We attempted to produce the requested figure (see below); however, as anticipated, it proved visually difficult to interpret due to the complexity and overlap of the data. Moreover, such a figure would only illustrate relationships along the first two principal components, which limits its interpretative value. Since it does not provide substantial additional insights, we prefer not to include it in the manuscript—unless reviewers or editors feel it is essential.

More importantly, if taxonomic grouping is one of the primary aims here, we think including all of the sediment spectra can be a potentially confounding factor. Why not explore PCA, clustering and LDA on fossil spectra alone? As noted above, this could also help shed more light on the role of preservation mode, skeletal mineralogy or even tissue chemistry.

→ See previous comments on the PCA and LDA performed with fossils only.

**Pseudoreplication.** The authors collected multiple spectra from each fossil/sediment specimen. We understand the rationale here, as any given spectrum could yield atypical results if it hit a specific contaminant etc., although it may be beneficial for the authors to elaborate a bit more on their reasoning in their methods section (ca. line 173-175) as well as how they selected their regions of interest. They mention choosing “seemingly homogenous regions” but it is unclear to me if they specifically targeted or avoided biomineralized phases like shells, scales and bones which might carry distinctive elemental signatures based on their original or altered mineralogies.

For exploratory purposes it is interesting to see how consistent/inconsistent spectra are from different portions of the same specimen (although this isn’t really addressed directly). It might be visually messy - but lines connecting the different spectra from the same specimen on Figure 4A could help illustrate variance within pseudoreplicates. This could be akin to a “phylogram” i.e. indicating some relational information between points with connecting lines. Again maybe this would be appropriate as supplementary information (the existing supplementary materials are not included with the submission or accessible at the Dryad link).

→ We are sorry. Indeed, there has been a problem with the Dryad link. This is now rectified. Regarding the identification of the pseudoreplicates in the figures, they are recognisable by being of the same colour. This has been clarified in the figure caption of Fig 4.

We do have a concern that the pseudoreplication might seriously inflate the LDA results. Have they considered generating a single mean or “consensus” spectrum for each specimen? The LDA is interesting as presented, but with the uneven taxonomic sampling and pseudoreplication it is difficult to say how easily this could be generalized to identify fossils of uncertain affinities.

Pseudoreplication could also skew cluster analysis, at least it would be interesting to consider whether samples from the same specimen are divided among clusters and if so, why this could be the case.

→ We understand the concern, but we respectfully disagree that pseudoreplication inflates the LDA results in this case. Actually, it is excluding the pseudoreplicates by averaging them into a single “consensus” spectrum per specimen and reducing the amount of data that would considerably artificially suppress within-group variance, and inflating the apparent separability of groups.

As mentioned previously, LDA functions by minimizing intra-group variance while maximizing inter-group variance. Including pseudoreplicates contributes meaningful within-group variability, which is essential for a robust and realistic assessment of group discrimination. Conversely, reducing each group to a single averaged point would limit variance. This would be all the more problematic given the uneven sampling across clades.

Additionally, our data show that even within a given specimen or anatomical region, there can be substantial geochemical variability. Including these pseudoreplicates captures this complexity and enhances the biological realism of the model. In this context, the use of pseudoreplicates does not artificially inflate the results but rather provides the necessary structure for a statistically and biologically meaningful analysis.

It would also be helpful to go into more detail about what is separating the clades in “chemo space”

→ We agree that understanding the specific chemical variables driving the discrimination between clades within a "chemo-space" would be highly valuable. However, LDA is not well suited for directly interpreting the contribution of individual variables, especially when it is applied to PCA-reduced data and across multiple groups. A detailed investigation into the chemical basis of group separation would require a different analytical approach and falls outside the scope of the present study.

That said, this question represents a logical and important next step, and we are currently working on a follow-up study to explore this aspect in greater depth.

**Phylogenetic/Taxonomic grouping.** The authors consider the taxonomic structure in their data across two different binning schemes and show that LDA can distinguish large-scale taxonomic groups within their dataset. However, it remains unclear whether a finer phylogenetic or taxonomic signal exists within certain groups, such as between actinopterygian and sarcopterygian fish (although the latter is only represented by a single sample so perhaps not much can be said) or among the diverse and well-sampled arthropod subclades. Furthermore, ammonoids and coleoids appear to follow distinct patterns in the LDA but this is unremarked upon in the text. More exploration of these internal taxonomic distinctions, and whether they map to phylogeny would strengthen the study's discussion and tie to the interesting ideas raised in lines 592-609. Again the authors may feel this more nuanced investigation is beyond the scope of the study but there appears to be a missed opportunity here. We recommend adding more detail about what is separating the clades in “chemo space”.

→ We understand this observation. As noted in the manuscript (e.g., LDA_1_; lines 647–648), the partitioning observed within the "chemo-space" may indeed extend to finer taxonomic scales. However, at this stage, the dataset is too limited to support statistically robust or empirically grounded conclusions at these levels. It is for this reason that we have focused our analysis on high-level clade groupings, excluding singletons. Such as to ensure the reliability of the statistical framework.

This study is intended primarily as a proof of concept, demonstrating that geochemical signatures can differentiate broad taxonomic groups. We agree that investigating finer-scale phylogenetic patterns represents a valuable next step, and we are actively working toward expanding the dataset to enable such analyses.

We question the need to include both Figs 8CD and 9AB as they show almost identical results despite the removal of one specimen, can you just show one or the other?

→ We agree, Fig 8C D has been removed.

Similarly Fig 5 does not show much that isn’t already visible from Fig 4, although it allows some additional insight into sediment patterns. As already noted a “fossil only” PCA (and cluster & LDA for that matter) would be a valuable addition.

→ We understand the concern, and we partly agree that the overall pattern in Figure 5 resembles that of Figure 4, albeit rotated. However, Figure 5 represents a separate PCA analysis conducted solely on the subset including sediment, echinoderms, and the two PPA samples. Prior to conducting this analysis, it was not possible to assume the structure would replicate that of the broader dataset. This subset PCA is the only statistically sound way to examine and interpret the relationships specific to these groups without the confounding influence of unrelated categories. For that reason, we believe that despite some visual redundancy, Figure 5 remains essential for a focused and valid interpretation of this subset and merits inclusion as a main figure.

**Line by line:**

Line 43: This is written as past tense but is still very much the case. We suggest clarifying the phrasing here along the lines of “Taxonomy in paleontology has historically relied on visual observations…”

→ Amended according to comment

Line 59: The phrasing here seems a bit off. Should it be “applied to imagery” rather than “applied for imagery” ?

→ Amended

Line 60: “a few peculiar elements” Again, the phrasing seems a bit odd, either explain in more detail which elements or perhaps just say. “This technique has even enabled the distinction…”

→ It was meant to be “particular”. Amended accordingly.

Line 85: Should “distant” be “distinct”? Or do you mean “widely distributed”?

→ In this context, we intended "distant" to refer to sites that are geographically far apart, rather than "distinct" in the sense of being different. While "widely distributed" is also appropriate, we opted for "distant" to avoid potential ambiguity, as "widely distributed" could be interpreted as referring to sites of large spatial extent rather than simply being far apart. We have therefore chosen to retain the original phrasing for clarity.

Figure 1/caption: Why is the LAK acronym not defined in the text or figure caption? Is it to preserve site confidentiality? Maybe I am missing explanation elsewhere, but it seems a bit odd to be left undefined, maybe explain or give a more explanatory abbreviation e.g. “UU” “unnamed Utah” or something along those lines?

→ Indeed, this site is still under study, as specified line 90-92: “We further add specimens from a new assemblage currently under study and located in northeastern Utah referred to here as LAK (Fig 1D; Brayard et al. ongoing work).”

Line 120: What are these eight clades? It would be helpful to enumerate them and tie them to the taxonomic list below since a variety of ranks and names are used in the materials list in 124–136. and on Figure 4

→ We agree. Amended as suggested

Line 154: Can you explain how the samples were prepped? Were they cut and polished?

→ The specimens were analysed without any additional mechanical preparation; they were neither cut nor polished prior to analysis.

Line 172–175: Can you elaborate a bit on the criteria used to pick and delimit each region? Were they selected based on highest/lowest apparent concentration of certain elements (that is sort of how it appears on Fig 2A). Are they all approximately the same area within or between specimens?

→ The regions were selected based primarily on a visual distinction between fossil material and surrounding sediment, without applying specific elemental criteria.

In cases where the fossil was poorly preserved (e.g., some ammonites), regions were limited in size due to the minimal amount of preserved shell material. Otherwise, regions were simply chosen large enough to obtain representative mean spectra with minimal noise.

Line 176–177: (two of them having been analysed during three experimental sessions). These turn out to be pretty important as you use them to tune your PCA analyses. Recommend removing this statement from parentheses and add some more detail: “Two specimens were analysed in all three experimental sessions in order to …” and then provide a bit of information about why these two were chosen, or if it was just arbitrary say that.

→ True, amended as suggested.

Similarly, do the samples which were scanned in each setup appear as duplicates on the PCA plots (Fig 3A-C) if so can they be labelled distinctly for reference on Fig 3A & 3B (e.g. with stars)? This would make for a helpful visual of the correction process. Presumably they are overlapping or nearly so on Fig 3C and subsequent PCA plots.

→ Fig 3C has been amended according to suggestion.

Line 193: suggest revising “detect from Phosphorus” to “detect elements ranging from Phosphorous…”

→ Amended as suggested

Line 196: Should “rang” be “range”?

→ Indeed

Line 197: Could you not just subset these points directly from the original spectra rather than the additional step of exporting the spectra as points and then reconstructing the spectrum? Maybe this is a function of instrumental outputs. Maybe you can clarify a bit here or in line 188?

→ Unfortunately, no, the spectra are necessarily exported as a succession of points, hence the continuous spectra reconstruction step.

Line 200: “Finally, all spectra were scaled through standardisation” What does this mean in practice

→ It means standardization strictly speaking, which is the adjustment of data so that it has a mean of 0 and a standard deviation of 1. Indeed, not to be confused with normalisation (rescaling data such as to fit a specific range). It has been specified such as to avoid this confusion.

Line 203: The authors use ‘morphometric’ and ‘morphology’ to describe the shape of the spectra and peaks, but this is potentially confusing because they are looking at chemical data and not fossil morphology. We recommend using a different term or clarify what is meant by the “morphometrics” of a uXRF spectrum. The procedure makes sense as described here but it might be valuable to the reader to more clearly underscore that “morphometric” is being used in a very different fashion from its typical use in paleontology.

→ Indeed, this terminology has been an issue throughout the whole writing process. However, after careful consideration, we have opted to retain this terminology, as we believe it most accurately conveys our approach. Nonetheless, in order to avoid as much as possible any confusion, we made sure to consistently associate these terms with the terms “spectra” or “spectral” in the body text.

Regarding the term “morphometric”, is was modified to “geometric morphometric”, and the section “Morphometric approach” was renamed “Geometric morphometric approach applied to the µXRF spectra”, in order to clarify our intended meaning and distinguish our usage from more conventional applications in palaeontology.

Line 211: Using just two specimens that were scanned across both setups as reference points to realign the entire dataset seems a bit risky, but this is a novel approach so for exploratory purposes this is OK.

→ It is true that only two specimens were scanned across both setups, however, each of these specimens contributed six spectral regions, resulting in a total of 12 reference spectra used for realignment.

Line 220: “effectively removing all setup specific morphological variations.” This seems like a bold statement without some type of independent cross-validation. The approach here seems reasonable but can you be confident that you “effectively removed all setup variation” with these corrections?

→ You are right; the original phrasing was too assertive at this stage. While the PCA results (Figure 3C) suggest that setup-specific morphological variations are no longer evident, PCA is a descriptive technique and does not provide inferential evidence to conclusively confirm their total removal. Therefore, we've revised the sentence to a more cautious formulation: “seemingly removing the vast majority, if not all, of the setup-specific morphological variations.”

Line 273: You have removed or at least reduced the influence of setup on your PCA results (clearly visible on PC1 of Fig 3A) however it seems difficult to exclude that there might still be some lingering influence in the overall PC scores, even though it is no longer the dominant feature visible on PC1 & 2 in Fig 3C. For example, the sediment samples for PUMA 2024 seem to separate from the other two analyses, especially at lower PC1 range although it is a subtle separation. not a major problem but be careful not to overstate.

Line 277: “there is no site-specific morphological distinction of the spectra (Fig 4A).”

Again, this seems like an overstatement. While the different sites don’t occupy distinct clusters, some sites appear to be restricted to certain portions of the PC1 vs. PC2 plot or to show site specific tendencies across these axes.

For example: STW samples show uniformly positive values along PC 2, and sediment values from this site  lie the extreme end of the sediment distribution in terms of PC1 (low) & PC 2 (high). Notably, the preservation at STW (carbonaceous) was different from most other samples (but carbonaceous shrimp from NN has similar PCA values and notably positive on PC2 scores).

IC may show opposite tendency at least for the sediments: PC 1 tends to be higher, PC 2 lower. LAK sediments and fossils all seem to plot along a shared PC1 v PC2 slope whose intercept appears to be shifted relative to the overall group. The best sampled site, PC, clearly seems to span a broad range and overlaps with everything else. But it is hard to tell if you can generalize this breadth to the other sites.

“Scores from our seven sites are broadly overlapping across the first two principal components, suggesting that site does not exert a dominant influence on uXRF spectra”

→ The statement has been rephrased to a more cautious formulation, according to suggestion.

Line 298: You defined “PPA” in your materials but it would be helpful for the reader to redefine at the first instance here.

→ Done

Line 300: “do not seem to present any specific arrangement in the PCA1 scatterplot”

Do you mean something more like

“do not seem to present any clear taxonomic (?) separation in the PCA1 scatterplot” ?

→ Yes. Amended according to suggestion

Although there aren’t clear, e.g., taxonomic clusters, and a good degree of scatter, there does appear to be some structure to the fossil data in the fossil+sediment analyses. Fossil samples do not scatter like a random cloud in PC1 v PC2.

Arthropod data appear to fall largely along two distinct slopes within PC1 v PC2 space. Site PC has samples that fall along both slopes but arthropods from other sites appear to be restricted to the “upper” slope (i.e., higher PC2 intercept). Generally, other taxonomic groupings appear to occupy relatively distinct areas/slopes on the PC 1 v 2 plot but this may reflect the small sample size and pseudoreplication from multiple samples from each specimen.

Line 302: “morphological gradient” can you clarify what this means in the context of your data? Presumably it implies that one or more elements vary continuously within these samples?

→ Exactly, although it is appropriate to deal about peaks rather than elements here. Now clarified in body text

Line 303: “echinoderm and spectra” do you mean “echinoderm and ammonoid spectra”?

→ Indeed

Line 306-321/Figure 5: We see the utility of this subset PCA but since it essentially reproduces the pattern well shown on Fig. 4 with a rotation, I’m not sure that Figure 5 provides much additional insight to merit inclusion as a main figure.

→ We understand the concern, and we partly agree that the overall pattern in Figure 5 resembles that of Figure 4, albeit rotated. However, Figure 5 represents a separate PCA analysis conducted solely on the subset including sediment, echinoderms, and the two PPA samples. Prior to conducting this analysis, it was not possible to assume the structure would replicate that of the broader dataset. This subset PCA is the only statistically sound way to examine and interpret the relationships specific to these groups without the confounding influence of unrelated categories. For that reason, we believe that despite some visual redundancy, Figure 5 remains essential for a focused and valid interpretation of this subset and merits inclusion as a main figure.

Line 343-345: “k-means clustering is one of the most commonly used clustering method, particularly due to its simplicity. However, it has many limitations, one of the main being that it assumes spherical clusters (68), which does not appear to be the case in our study. Therefore, the”

This reads like discussion. We suggest just to report the result here:

Something like: “When constrained to two groups, K-means clustering failed to separate fossils from sediments.”

→ We agree. We have revised the sentence to focus on the result. However, given that not all readers may be familiar with the limitations of k-means clustering, we included a brief clarification to aid comprehension.

Line 359–360: Did you make thin sections to see if sponge spicules or other microscopic biomineralized materials occur within sediments?

→ Thin sections have been studied in the past, and no clear evidence of abundant biomineralized material has been observed in the sediment.

Line 379 vs. 385:

“relatively high taxonomic resolution” “

“but this time at the high-level clade resolution”

These describe two taxonomic binning levels: 9 groups vs. 13 groups but the phrasing here is too similar. Can you contrast them more clearly?

Maybe “relatively fine taxonomic resolution” to characterize the 13 group scheme?

→ Of course, amended according to suggestions

Line 403/Fig 9

While this was a good idea, it had very limited impact on the overall results (at least for the LDA), we are not sure it needs to be reported in the level of detail here but to note that the overall results were essentially the same and direct to supplement if needed. Likewise Fig 9A/B essentially duplicate Fig 8C/D and don’t need to be included.

→ Indeed, this might be a bit repetitive. Therefore, the Fig.8 C/D was moved to supplementary material. We favour to keep LDA_3_ (without singleton), because it is the most robust, statistically speaking.

Line 416: - You switch to PCA3 (i.e. sarcopterygian excluded) and focus on this for the remainder of the manuscript. Why when the exclusion of the sarcopterygian had such limited impact? It is not too big of a deal either way but it is a bit confusing when all of the results presented in Figs 4, 6–8 show PCA1.

→ We switch to PCA_3_ because it is the data used for LDA, that beyond being the most effective, is the most statistically robust.

Line 460: And notably fell along a shared slope with sediments. Is this due to a shared carbonate composition? Probably worth stating this outright if you think that is correct.

→ Right. Amended following reviewers’ advice

Line 465: “no apparent mineralogy-specific morphological signature among the spectra of the fossils studied here”

Again, it doesn’t emerge as a dominant factor in the various analyses, but also this wasn’t really framed as a hypothesis earlier in the paper and most of the analyses and figures don’t really seem to show or address mineralogy as a primary factor so it is hard to know what factor it might be playing here.

→ See answer to general comments

507 - what do you mean by multi-sampling here? Studying multiple samples from the same locality/horizon, or multiple samples from the same specimen (as you also do here)? How much variation along PC1 is observed when considering sediment spectra from the same specimen?

→ Here we mean multiple samples from the same locality/horizon. Amended to clarify: “The existence of this geochemical gradient within the sediments shows that even within a same site and stratigraphic horizon, the geochemistry of the sediment is variable, underscoring the importance of collecting multiple sediment samples within a single locality and stratigraphic horizon when conducting geochemical studies

Line 507-513

The lithology of most (all?) specimens is calcareous shale, so the Ca (and Sr) are to be expected but the more detrital (silt, clay) material you have, the carbonate fraction is reduced relative to these Fe/Zn bearing phases. Wouldn’t that explain the contrast that you see? Weathering would exacerbate this as carbonate is more soluble, and an organic matter/microbial seems a plausible contributing or alternative mechanism but seems more elaborate than needed.

→ Right, that is what we suggest here: “weathering processes that could have very locally (e.g. centimetres to metres) and differentially dissolved the calcium, leading to a relative increase in iron concentration”

Line 513-517: OK well put, but you could state this more clearly earlier on in the text (see comments above).

Line 527-528: Yes as you noted on Line 126 these are primarily molds so we would probably expect them to look like sediments, but the fact that they overlap with echinoderms suggests more calcitic (recrystallized?) shell material than expected?

→ Yes, relatively more compared to sediment only. That’s the idea.

 Line 541: “very disparate sizes, with the smallest one (i.e. sarcopterygian) being represented only by one specimen”

Suggest adding “sample” before “sizes” to clarify what you are talking about here, “size” can mean many different things in this context.

→ You are right. Amended accordingly

Line 552: “high intra-clade preservation variability” Some clades have multiple preservation modes (arthropods, fish, brachiopods although in the case of the latter 2, even the ‘carbonaceous’ fossils likely retained some of the phosphatic shell/bone material) other clades only show one mode of preservation. Recommend toning this down to: “ specimens with variable preservation and from different distant localities” \

→ Yes. Amended following reviewers’ advice

Line 553-555: Did you consider including any indeterminant specimens to test this?

→ This is the next step, and ongoing work

Line 557: Why save all this for discussion? Identification of key elemental peaks associated with sediments or fossils seems appropriate for results.

→ We saved this for the discussion because one of the key aspects of the methodology presented in the manuscript is to defer interpretation of elemental contributions to later stages, and base it on empirical data

Line 576-578: Based on Fig 10 - The results here seem to really mirror what is reported earlier - probably because the trend identified on PC axis 1 (essentially Ca/Sr vs. Fe and other transition metals) is so strong, especially on the sediments and calcitic fossils.

→ Yes and no. There are elements that contribute as expected, and seemingly obvious just looking at PC axis 1, but then there are others that are more important than expected (e.g. K, Ce, Nd, Ni, Th), or conversely less (e.g. V, Cr, Co, Zn, Br, Sr)

The entrance of potassium here as a new player is of interest given that it is a key nutrient but goes unremarked upon here.

Line 564-565: “most likely as a marker of echinoderm and ammonoid fossils.”

Is there any question that this is the case?

It certainly appears this way on e.g., Figs 8A,C; 9A which broadly distinguish calcitic taxa & sediments from non calcitic taxa.

→ Indeed, not really. Amended to “most certainly” in body text

It does appear that the LDA is able to “pull the PPAs and the better preserved ammonoid into a tighter cluster than the PCAs themselves show, but this isn’t discussed. Any idea of what’s going on here?

→ We agree that the LDA appears to group the PPAs and the better-preserved ammonoids more tightly than the PCA. This likely reflects the fact that LDA, being a supervised method, is able to emphasize subtle differences between predefined groups by maximizing between-group variance relative to within-group variance. In contrast, PCA captures the directions of highest variance in the data overall, without reference to group structure. Unfortunately, the LDA does not correctly allow to trace back the specific spectral features responsible for this clustering.

Incidentally the gladii of the two coleoids were presumably primarily calcitic/aragonitic but plot quite far from the calcitic fossils on LD1 (although interesting seem to show similar LD2 values). Did the microXRF scans include any of the gladius material? Apparently the phosphatic preservation is overprinting the calcitic signal on LD1 but why do the echinoderms and cephalopods show similar LD2 values relative to everything else.

→ Unfortunately, although LDA is a powerful tool for discriminating between predefined groups, given its functioning it does not readily allow for direct interpretation of the underlying variables, especially when it is performed on PCA scores rather than on raw spectral data, and with many groups. Similar or dissimilar LD values could be the result of a single or many, small or big morphological variations, unidentifiable directly for now. One of the next steps is indeed to clearly characterise the elements that distinguish each taxonomic group from each other, and it is an ongoing work.

Line 576-578: Not sure that you need to list all of the minor elements that don’t contribute to discrimination unless there is some reason why this is of particular interest?

→ We agree that listing all minor elements may seem excessive, but our intention here is twofold: (1) to highlight which elements contribute meaningfully to group discrimination in our dataset, and (2) to identify those that appear uninformative under these conditions. We believe this information could be useful for future studies aiming to optimize analytical focus or streamline data collection.

**Figure 4 -** It would be helpful to note the total number of specimens belonging to each taxon next to the legend labels, so: Echinoderms (n=6) etc.

Figs. 4A, 8A etc. Do you need the “gladius-bearing” modifier in the legend? For simplicity can you just call these “coleoids?”

Using symbols or colors to show different preservation modes (carbonaceous, phosphatized, calcitic) on PCA plots would provide an interesting complement (maybe more interesting than symbol by site although it would be worthwhile to show both).

→ As previously explored, we attempted to represent preservation modes using different symbols or colors on the PCA plots. However, due to the number of overlapping data points and the complexity already present in the plots, the result was visually cluttered and difficult to interpret. In practice, this representation did not add meaningful clarity or insight and was ultimately not informative. For this reason, we opted not to include such a figure in the manuscript.

4B & C, it might be helpful to label key peaks here even though representative elements are shown on Figure 10.

→ Amended as recommended

**Figure 5 -** As noted above, Fig 5A this does not provide a great deal of clarity beyond what was shown in Figure 4 and could probably be moved to the supplement. If you keep it, we again suggest labelling key peaks in Figure 5B as suggested for Figure 4.

→ Amended as recommended

**Figure 7 -** Can you include the taxonomic/site color & shape legend here? Also, removing the pseudoreplication of specimens could allow for more interesting trends to emerge.

→ Regarding the inclusion of the taxonomic/site colour & shape, theoretically we could yes, but it would not be make much sense as this figure is meant to illustrate the LDA and its discrimination.

Regarding the pseudoreplicat issue, please refer to answer above.

**Figure 8** - Legends use a mix of technical and common names, singular/plural (wethink all are represented by multiple specimens except the one sarcopterygian?). Common names might be the most reader-friendly option if they are unambiguous (so Actinopterygians could be Ray-finned fish etc.) but either sticking with common names or scientific names seems preferable to a mix of the n=ntwo.

→ Amended as recommended

**Answers to reviewer 2:**

Here, Smith and coauthors present a suite of synchrotron beam x-ray fluorescence (XRF) data to explore elemental signatures of taxa across a suite of Paris Biota fossils from the Western USA. The authors use multivariate methods to compare full XRF spectra across the sample set. They show distinct differences in compositions at the clade level and some regional similarity in sediment/diagenetic composition. This work is interesting; however, it requires some revision to highlight the utility of this approach and to situate it better in the context of preexisting literature as it stands right now it may be overselling novelty and not highlighting the actual utility. I am recommending what may end up being some substantial revisions to the text, but support this work as being interesting and important to show both new analytical techniques and a useful statistical framework that circumvents some problems of the analytical technique.

1. Unclear how this is distinct from existing broad clade level distinction of biominerals? You already cite works like (Ulrich et al., 2021).

→ Given the age of the Paris Biota specimens, the spectral differences we identify are unlikely to reflect pristine biomineral composition alone. Instead, our results suggest the presence of a deeper, clade-linked geochemical signature, preserved through diagenesis and time, that can be distinguished via whole-spectrum analysis. This represents a significant advancement beyond traditional biomineral-based classification of modern or sub-modern specimens. Additionally, this goes beyond the simple type of preservation/original tissue nature of the fossils, as we have specimens of the same clade presenting various mineralogical nature/type of preservation and also specimens of different clades with similar mineralogical nature/type of preservation. We believe this distinction is already stated in lines 38–40 and 73–74, but we have now clarified it further with minor textual amendments.

It seems that you are implying that debates like the affinity of Tullimonstrum can be solved with this clade-specific geochemical fingerprint, but at the phylum level this is too coarse to be useful, I think? This was the area that was most confusing to me because it seems like you are proposing a solution for a problem, but it is unclear how useful it can be in practice outside of a single formation where you have to calibrate with morphological identification then geochemical characterization and then finally unknowns could be classified.

→ We understand the reviewer’s concern. Our approach is for now only restricted to the Paris Biota and at a high-level clade resolution Our objective is to demonstrate, as a proof of concept, that a clade-specific geochemical signal can persist even in diagenetically altered fossils, and that the latter may serve as a new taxonomic tool. This work is a necessary first step toward testing broader applicability, the limits of which are not yet defined and are part of ongoing research.

2. There is an interesting note of possible diagenetic pathways, but this could use some expansion. For example in cephalopods show the soft tissue differences that cause dramatic differences in preservation (Clements et al., 2017).

→ Indeed, that aspect had not been noted. It is now added lines 612-613.

3. Limiting this work to synchrotron x-ray sources means that it is not widely accessible. Is the goal application to small shelly fauna or Cambrian enigmas? Is it something that can be done with a handheld XRF or tabletop XRF or even EDS spectra given mapping across a surface? There is not a clear way to expand this work as it is currently presented to the readers.

→ Indeed, the potential for broader application, albeit in a more exploratory capacity, is already addressed in lines 674–684. This paragraph clarifies that while the current model is trained on the Paris Biota, the approach could be extended to many other fossil assemblages and geological contexts. Furthermore, although synchrotron data were used in this study, the method is not inherently restricted to such facilities. As long as complete spectral data are available, techniques such as benchtop µXRF or SEM-EDS may serve as viable alternatives. We emphasize that our goal was to demonstrate the potential of this method in an exploratory context, laying the groundwork for future adaptation, including to more easily accessible lab platforms.

4. I know it is beyond what you can do in this study, but to say you have a taxonomic discrimination tool that is independent of a priori taxonomic distinction without a broader dataset including modern specimens feels poorly supported. You can adjust by softening language probably or framing things as future research direction.

→ There may be a misunderstanding. Our taxonomic discrimination tool is specifically designed for use on fossil specimens, not on modern, pristine biominerals. Given that these fossils have undergone diverse diagenetic processes, their XRF spectra are fundamentally different from those of modern specimens. As such, comparing them directly would not be meaningful, as they represent two distinct objects. Nonetheless, we agree that exploring how this approach might extend to modern taxa or be calibrated against modern analogues could be a valuable direction for future research.

In response to comments from both reviewers, we have also softened the language throughout the manuscript, particularly in relation to the apparent absence of site and mineralogical effects.

5. You state that site specific clusters do not exist, but I don’t think I see any visualization of the points coded by site after you standardize them. It would be useful to see this

→ We respectfully note that the site identity is already represented in our visualizations In Figure 4A (PCA of all data after standardization), Figure 5A (PCA of sediment, echinoderms, and two PPA after standardization), and Figure 7 (hierarchical clustering), the shapes of the data points highlight the different sites.

**Line by line:**

Lines 55-59: Much of the capabilities of micro x-ray fluorescence recapitulates some of the capabilities of electron microprobe mapping across fossil materials? I agree prep is easier and throughput may be higher, but this seems to be less grounded in informing readers of the history of the field of elemental mapping across fossils

→ Yes, you are right. Amended such as to refer to electron microprobe and PIXE as the grounding of µXRF and present a bit better the evolution of the techniques

Lines 66-68: I think the deconvolution tools are likely and important thing to expand on here and in the discussion.

Lines 77-78: Mineralogical variability may be vague here. Variation in mineral implies calcite or aragonite or vaterite?

→ Here “mineralogical variability” refers to that of the fossils, and not the original mineralogy. In a sense, the type of preservation (e.g., some shrimps pyritised while others are phosphatised). Clarified by specifying “fossils intra-clade mineralogical variability”

Line 123: Dryad data was a dead link when I tried to use it.

→ We are sorry. The previous link has been modified to a full temporary reviewer sharing link: http://datadryad.org/share/EFH1rI439erErLsdvLdpcxyuutUgKHwfmr3dj61BmaE

Line 125-139: Make this a table for quick comparison.

→ As mentioned in the body text, a table is available in supplementary material. It is not directly included in the manuscript as it is already dense. It can however be included in the manuscript if reviewers deem it necessary

Line 126: Although the overall sample may have been a mold if you sample the shell with the XRF why the distinction between them?

→ It is because given the little amount of shell preserved, we cannot exclude that sediment has also been sampled.

Line 171-174: Was there a minimum area? What was the criteria for seemingly homogeneous? Why not just randomly subsample each area algorithmically and integrate that whole randomization procedure into what you’ve done? How does this step not add user-bias?

→ Some specimens presenting only a very little amount of “shell” (e.g., PPA), there wasn’t any minimum area. However, when possible, the areas were selected wide enough to have “clean” mean spectra (without noise). Seemingly homogeneous is simply visually distinguishing between fossil and sediment. We did think about algorithmically subsampling, however that would have required some elemental criteria common to all the specimens to distinguish fossil from sediment, which would have clearly biased the analysis. The selected areas may be found in the supplementary material S1 – Data S1.

Line 200: Standardization meaning scaling peak to lowest count the same across spectra?

→ Here it is a classic standardization (strictly speaking) in the sense of adjusting data so that it has a mean of 0 and a standard deviation of 1. Indeed, not to be confused with normalisation (rescaling data such as to fit a specific range). It has been specified such as to avoid this confusion.

Line 206: The 831 landmarks presumably align with how many possible elements and their X-ray signature in your actual sample?

→ The landmarks describe the full µXRF spectra. The setups having been optimised in order to cover from Phosphorus, with Kα1 = 2.146 keV, to Uranium with L3 = 17.166 keV, the landmarks cover all elements that may present a peak between those bounds. However, a single element can also present multiple peaks (K_i_, L_i_, M_i_), so it is hard to quantify how many potential signatures in total.

Lines 281-282: Why not just provide a most likely element for each peak?

→ This is because the aim here is to study the overall morphology of the spectra without making any assumptions about the presence or absence of specific elements. Elemental interpretation comes afterward, through empirical and statistical validation, allowing us to detect subtle yet significant features that are likely invisible to the naked eye.

Line 387: Internal gladius vs external shell are not mineralogical distinctions. Ammonites have shells made of the mineral aragonite and gladius composition is likely to also be aragonite, but may be comprised originally of chitin (Doguzhaeva and Mutvei, 2006).

→ We agree, “nature” is more correct. Amended accordingly.

Lines 466-467: I am confused about this statement. What does a mineralogy specific bias here mean? That aragonite or calcite or vaterite create secondary structure in the XRF spectra? Or do you mean that diagenetic alteration from aragonite to calcite does not drive any bias?

→ A mineralogy-specific bias would mean that the morphological differences of the spectra are primarily driven by their mineralogical nature, rather than by differences in taxonomy, preservation quality, or other factors. Here we show that the spectral differences between fossils do not appear to systematically follow their mineralogical composition i.e., the morphology of fossil spectra is not dominated or skewed by mineralogical nature. Modified to clarify this point.

Lines 654 and 663: You use user-bias-free and setup-specific bias in these two sentences and I don’t quite follow the distinction. Is the user-bias an interpretive bias (which is still present given the need to explore clustering algorithms) or is it a setup-bias associated with instrumentation?

→ The term “user-bias-free” refers specifically to the taxonomic identification process, which is inherently interpretive, and thus, at least partially subjective. In contrast, the approach presented here is empirical and statistically based, and therefore more objective. Even the clustering, while multiple algorithms are tested to determine the most effective one, remains an empirical process rather than a subjective interpretation. That said, as the term “user-bias-free” may come across as overly assertive, it has been removed.

Regarding the term “setup-specific bias”, it indeed refers to instrumentation, consistent with how it is phrased elsewhere in the manuscript.

**Figure comments**

Figure 3. It would be useful if you include annotations for the element peaks associated with energy. Also, why do you not report or attempt to report the weights of the spectra on each PCA axis either with a simplification of using 1 or two energies per element to show the general discrimination? It would be a simple way to ground your discussion of the interpretation of the trends in multivariate space which you bring back to the elemental compositions anyway in your discussion.

→ We acknowledge the usefulness of including annotations indicating the elements corresponding to each peak in Figures 3D–E. We attempted this. However, due to the large number of peaks, the resulting figure became overly cluttered and difficult to interpret. Given that one of the key aspects of the methodology presented in the manuscript is to defer interpretation of elemental contributions to later stages (based on empirical observations), we believe it is more appropriate to keep Figure 3 focused on illustrating the spectral readjustment. As this figure appears early in the manuscript and serves a different illustrative purpose, we prefer to retain its current form for the sake of clarity and visual readability. We however added a few major elements to the other illustrated spectra (Figures 4C-D, 5B, 9C-D) to make it easier for readers to interpret the spectrum.

In order to better visualize the morphological variation represented by the principal components of PCA0 and PCA1’, a figure representing the morphology of the spectra at the positive and negative poles of the two first principal components of PCA0 and PCA1’ was indeed lacking in the initial version of the manuscript. The Figures 3A-C containing already a lot of information, we prefer to retain their current form. However, we added to the supplementary material, a figure representing the morphology of the spectra at the positive and negative poles of the two first principal components of PCA_0_ and PCA_1’_.

Figure 4. Same comment on showing the elemental weights on the axes as in Figure 3. The sliding scale of color within types (e.g. sediment color gradient) is distracting, although I assume you did it to show distinctions between the points? Adjust alpha uniformly if overplotting is what you’re worried about. Each change in the visualization (e.g. x/y/color/symbol) should be data that can be interpreted.

→ We do not understand the first part of the comment regarding the elemental weights of the axes in PCA. We believe there may be a misunderstanding. The PCA in this study was conducted on the morphology of the µXRF spectra, not on elemental concentrations. As such, the principal components do not correspond to elemental weights, but rather represent gradients in spectral morphology. To illustrate these gradients, we show the average spectral morphology at the positive and negative extremes of each principal component (see Figure 4B–C).

Regarding the sliding scale, this is to distinguish the different samples rather than the points, with all pseudo-replicates of the same sample being of the same colour. Clarification of this was added to the figure caption of Figure 4.

Figure 9. Why are A and B in this replicate of C and D in Figure 8 with B being mirrored on the X axis? Why did the format of the x axis text in the spectra change so dramatically compared to the consistent constraints of the earlier figures? What do the background colors on the spectra mean in C and D and are they telling us anything other than what is visible with the positive or negative pole curve

→ Figure 9A-B and Figure 8C-D are different and do not illustrate the same analysis. Figure 9A-B is the LDA without singleton (no sarcopterygian), whereas Figure 8C-D integrates all data. However, these figures being a bit repetitive, Figure 8C-D was moved to supplementary information.

The x axis text has been homogenised in all figures.

Figure 9C-D being temperature diagrams, the background colours represent the relative contribution of each spectral region to the overall discrimination observed in LDA_3_. Regions shaded in red indicate areas of the spectra that contribute most strongly to the discrimination, while lighter-coloured regions correspond to lower contributions. To clarify the direction of the colour gradient, it has been added to the Figure 9 caption.

Figure 10. Again, why is the x axis formatted inconsistently (values, energy Kev) compared to earlier figures?

→ As noted previously, the x axis text has been homogenised in all figures.

Thank you, and please feel free to contact us for any additional inquiry,

Best regards,

Christopher P. A. Smith & co-authors
